# Draft Genome Sequence of *Pseudoalteromonas tetraodonis* Strain MQS005, a Bacterium with Potential Quorum-Sensing Regulation

**DOI:** 10.1128/genomeA.00724-16

**Published:** 2016-08-04

**Authors:** Yonglong Pan, Yanbo Wang, Xiaoqing Yan, Asit Mazumder, Yan Liang

**Affiliations:** a Shenzhen Institutes of Advanced Technology, Chinese Academy of Sciences, Shenzhen, People’s Republic of China; b Jilin University, Jilin, People’s Republic of China; c University of Victoria, Victoria, British Columbia, Canada

## Abstract

We present here the draft genome sequence of *Pseudoalteromonas tetraodonis* strain MQS005, a bacterium possessing potential quorum-sensing regulatory activity. This strain was isolated from water from the South China Sea, People’s Republic of China. The assembly consists of 4,252,538 bp and contains 144 contigs, with a G+C content of 41.85%.

## GENOME ANNOUNCEMENT

*Pseudoalteromonas tetraodonis* strain MQS005 was isolated from water from the South China Sea, People’s Republic of China. *P. tetraodonis*, a deep-sea marine bacterium, has a symbiotic relationship with fish (e.g., pufferfish) and produces neurotoxin tetrodotoxin (1). We found that this strain could efficiently degrade the quorum-sensing (2) signaling molecules acyl-homoserine lactones (AHLs) and secrete long-chain AHLs upon short-chain AHL induction.

Genomic DNA of *P. tetraodonis* strain MQS005 was extracted using the TIANamp bacteria DNA kit (Cat. no. DP302; Tiangen Biotech Co., Ltd., Beijing, China). A paired-end library with an insert size of 350 bp was constructed and sequenced using the Illumina HiSeq platform with a PE150 strategy. Then, the high-quality reads without PCR adapter sequences were assembled by SOAP*denovo* (3) to generate scaffolds, and all reads were used for further gap closure. A total of 1,214 Mb of high-quality sequence data were obtained, and the resulting assembly consisted of 4,252,538 bp containing 144 contigs (longest, 221,568 bp; *N*_50_, 60,836 bp), with a G+C content of 41.85%. With gene prediction, up to 3,943 genes in the genome assembly of *P. tetraodonis* strain MQS005 were found by GeneMarkS (http://topaz.gatech.edu/) with integrated model and Heuristic model parameters. Gene annotation was performed with a Blast search (http://blast.ncbi.nlm.nih.gov/) (*E* value <1e^-5^ and minimal alignment length larger than 40%) against 4 databases. A total of 3,738, 2,222, 1,977, and 2,692 functional genes were annotated from the Kyoto Encyclopedia of Genes and Genomes (KEGG) (4), Clusters of Orthologous Groups (COG) (5), Nonredundant protein (NR) databases, and Gene Ontology (GO) (6), respectively.

### Accession number(s).

The genome sequence of *P. tetraodonis* strain MQS005 was deposited at DDBJ/EMBL/GenBank under the accession no. LYRQ00000000. The version described in this paper is version LYRQ01000000.
